# Genetic variation in innate immune gene expression influences mortality after traumatic brain injury in *Drosophila*

**DOI:** 10.1093/g3journal/jkag060

**Published:** 2026-03-13

**Authors:** Rebeccah J Katzenberger, Nathaniel P Sharp, Barry Ganetzky, David A Wassarman

**Affiliations:** Department of Medical Genetics, School of Medicine and Public Health, University of Wisconsin-Madison, Madison, WI 53706, United States; Department of Genetics, College of Agricultural and Life Sciences, University of Wisconsin-Madison, Madison, WI 53706, United States; Department of Genetics, College of Agricultural and Life Sciences, University of Wisconsin-Madison, Madison, WI 53706, United States; Department of Medical Genetics, School of Medicine and Public Health, University of Wisconsin-Madison, Madison, WI 53706, United States

**Keywords:** age, animalia, antimicrobial peptide, diet, *Drosophila melanogaster*, Imd, innate immunity, NF-κB, toll, traumatic brain injury

## Abstract

Traumatic brain injury (TBI) is a leading cause of disability and death, with outcome severity varying widely even among individuals with comparable injuries. A major challenge is to identify pathways that underlie this variation and could be targeted to improve therapies. Innate immune pathways are candidates because they are rapidly activated after TBI and contribute to neurodegenerative disorders. Using a *Drosophila melanogaster* TBI model, we examined how genetic background, age, and diet modify effects of evolutionarily conserved Toll and Immune deficiency (Imd) pathways on injury outcomes. These pathways signal through nuclear factor-kappa B (NF-κB) transcription factors Dorsal-related immunity factor (Dif) and Relish (Rel) to activate antimicrobial peptide (AMP) gene expression. We found that genetic diversity among lines from the Drosophila Genetic Reference Panel (DGRP) contributed to variation in AMP expression before and after TBI, with additional effects of age and diet. AMP expression tended to be correlated positively with early mortality following TBI in young flies, but negatively in older flies, suggesting an age-dependent shift in AMP effects from detrimental to protective. Furthermore, heterozygous mutations in *Dif* or *Rel* lowered AMP expression in a diet-dependent manner and led to correspondingly reduced early mortality after TBI. These findings show that genetic, biological, and environmental factors influence innate immune pathways, which in turn determine TBI outcomes. Innate immune gene expression before injury emerges as a potential prognostic indicator, pointing to potential new therapeutic strategies.

## Introduction

Traumatic brain injury (TBI) resulting from a blow to the head or body affects an estimated 70 million people worldwide each year (Dewan et al. 2018). However, progress in improving prognosis and treatment remains limited (Kawata et al. 2024; Imberti 2025). One of the most pressing and unresolved questions in TBI research is why patients with comparable injuries often experience substantially different outcomes (O’Brien et al. 2020; McCrea et al. 2021). Addressing this fundamental challenge is critical, as it points to underlying biological mechanisms that could be targeted to improve prognosis and enable precision therapies.

Innate immune signaling in humans appears to be a major determinant of TBI outcomes (Bouras et al. 2022). After injury, damaged cells release damage-associated molecular patterns (DAMPs), which activate Toll-like receptors (TLRs) (Baral and Kaundal 2025). TLR activation triggers cytokine production that can limit cell damage and promote repair but may also contribute to oxidative stress, edema, excitotoxicity, and neuronal death in the brain. Consistent with this, mice lacking TLR2 alone or both TLR2 and TLR4 show milder TBI outcomes, highlighting the detrimental role of TLR signaling (Yu and Zha 2012; Krieg et al. 2017). However, most rodent studies test TLR pathways under a single experimental condition, without accounting for genetic background, age, and diet (Marklund 2016)—factors that differ among TBI patients and likely influence immune responses as well as pathophysiological outcomes (Treble-Barna et al. 2020; Cortes and Pera 2021; Delage et al. 2021; Lu et al. 2026). Such factors may influence which genes are regulated by TLR pathways, their transcription levels, and the specific cells in which they are transcribed. Defining these modifiers and how they influence innate immunity after TBI is essential for developing therapies that are broadly effective across the spectrum of patient populations.

*Drosophila melanogaster* is an excellent model for studying how innate immunity influences TBI outcomes due to its simplified set of receptors. While rodents have 12 TLRs involved in innate immunity (Baral and Kaundal 2025), *Drosophila* encodes nine Toll receptors but relies primarily on Toll-1 (Toll) for most immune responses (Valanne et al. 2022; Hanson et al. 2025). DAMPs also activate the fly immune deficiency (Imd) pathway, homologous to the mammalian tumor-necrosis factor receptor (TNFR) pathway, which plays a neuroprotective role in mouse TBI models (Sullivan et al. 1999; Yang et al. 2010; Bier and Guichard 2012). Toll and Imd signaling activate nuclear factor-kappa B (NF-κB) transcription factors, Dorsal-related immunity factor (Dif), and Relish (Rel), respectively, which drive expression of genes such as antimicrobial peptides (AMPs) that mediate the inflammatory response (De Gregorio et al. 2002; Lemaitre and Hoffmann 2007; Ganesan et al. 2011; Lee et al. 2024). Similarly, in mammals, NF-κB family members act downstream of TLRs and TNFR to induce cytokine gene transcription in response to DAMPs (Mettang et al. 2018; Bouras et al. 2022). In addition to their simplified and conserved innate immune pathways, *Drosophila* offer the advantage of rapid, low-cost testing of multiple experimental conditions, allowing the screening of genetic and environmental factors that may modify effects of innate immunity on TBI outcomes (Bilen and Bonini 2005; Oriel and Lasko 2018).

Using a high-impact trauma (HIT) device to induce TBI in flies (Katzenberger et al. 2013, 2015b), we found that innate immune genes, including AMPs, were the main class of genes upregulated in *w^1118^* flies (a standard laboratory fly line) at 4 h after injury (Katzenberger et al. 2016). Expression of AMPs increased within 30 min post-injury and remained elevated for at least 24 h, indicating that TBI rapidly and persistently activates the Toll and Imd pathways, analogous to TLR and TNFR pathway activation by TBI in mammals (Bouras et al. 2022; Baral and Kaundal 2025). The strength of this response varied across four age-diet conditions: (1) young flies (0–7 days old) fed a standard high-carbohydrate cornmeal-molasses-yeast diet (CMYD), (2) young flies fed water only, (3) old flies (20–27 days old) fed CMYD, and (4) old flies fed water only (Katzenberger et al. 2016). We also found that a heterozygous mutation in *Rel* reduced early mortality (within 24 h post-injury) in young flies fed CMYD, although the magnitude of this effect varied across genetic backgrounds represented in the Drosophila Genetic Reference Panel (DGRP), a collection of genetically diverse inbred lines derived from a wild population (Mackay et al. 2012; Swanson et al. 2020b). In addition, mutation of the AMP gene *Metchnikowin* (*Mtk*), but not six other AMP genes, reduced early mortality in young flies fed CMYD (Swanson et al. 2020a). Together, these findings demonstrate that the fly TBI model captures the importance of innate immunity observed in mammalian TBI and provide evidence that genetic background, age, and diet modify how NF-κB pathways influence TBI outcomes.

Building on these findings, we examined in greater detail how genetic background, age, and diet influence innate immunity and its impact on TBI outcomes. We measured the expression of nine AMP genes and early mortality in 19 DGRP lines across the same four age-diet conditions, as well as in *Rel* mutants and newly generated *Dif* mutants. These studies show that genetic background, age, and diet interact in complex ways to influence innate immunity and early mortality, which may help explain the variation in TBI outcomes observed in humans.

## Materials and methods

### Fly lines and culturing

Flies were maintained on a standard fly food, cornmeal-molasses-yeast diet (CMYD), at 25 °C. CMYD contained 30 g Difco granulated agar (Becton-Dickinson), 44 g YSC-1 yeast (Sigma), 328 g cornmeal (Lab Scientific), 400 ml unsulfured Grandma's molasses (Lab Scientific), 3.6 L water, 40 ml propionic acid (Sigma), and tegosept (8g methyl-4-hydroxybenzoate in 75 ml of 95% ethanol) (Sigma) (Blommer et al. 2021). Water vials were prepared immediately before use by placing a circular piece of Whatman filter paper (GE Healthcare Bio-Sciences) at the bottom of the vial to absorb 200 μl of water. *w^1118^* flies were maintained in our lab for many years, and the 19 randomly selected DGRP lines (Mackay et al. 2012) were obtained from the Bloomington Drosophila Stock Center (DGRP26, DGRP73, DGRP91, DGRP93, DGRP161, DGRP352, DGRP381, DGRP382, DGRP383, DGRP385, DGRP386, DGRP409, DGRP427, DGRP439, DGRP440, DGRP441, DGRP774, DGRP892, and DGRP897).

### Generation of *Dif^del^* and flies heterozygous for *Dif^del^*, *Rel^del^*, and both mutations

A *Dif* null allele was generated by CRISPR-Cas9 homology directed repair using the pHD-DsRed-attP vector (Addgene, Plasmid #51019) as the donor template (Gratz et al. 2014); 1 kb left and right homology arms were generated immediately flanking *Dif* cleavage sites upstream of the transcription start site and downstream of the transcription termination site. Homology arms were amplified via PCR with 20 bp overhangs homologous to the pHD-DsRed-attP vector, and the vector was amplified via PCR with 20 bp overhangs identical to the homology arms. PCR products were purified with the Wizard SV Gel and PCR cleanup system (Promega). The donor plasmid was constructed by Gibson assembly of the four DNA fragments (Gibson et al. 2009). Synthetic guide RNA (sgRNA) plasmids were generated using the pU6-3 gRNA vector generously provided by the O’Connor-Giles lab (Brown University). Guide sites were selected using flyCRISPR optimal target finder (Gratz et al. 2014). Both sgRNAs had no predicted off-targets. sgRNA plasmids were constructed by amplifying sgRNAs and the pU6-3 gRNA vector by PCR with 20 bp overhangs. PCR products were purified with the Wizard SV Gel and PCR cleanup system. sgRNA plasmids were assembled using the Kinase Ligase Dpn (KLD) mix (New England Biolabs). sgRNA plasmid sequences were confirmed by sequencing. Plasmid DNA was prepared for injection by a mini- or midi-prep kit (Qiagen) and was injected into vas-Cas9(II) embryos (Bloomington Drosophila Stock Center, Stock #56552) by BestGene. Genetically identical control lines were developed from the injection stock. Positive transformants were identified through the fluorescent DsRed eye marker, PCR, and qRT-PCR. Primer sequences are in Supplementary Table 1. Cas9 was removed from the stocks by backcrossing eight times to the injection stock that did not contain Cas9. The same approach was used to generate *Rel^de^*^l^ flies (Swanson et al. 2020b).

Heterozygous *Dif^del^* flies were generated by crossing *Dif^del^*/*Dif^del^* flies to genetic background-matched control +/+ flies. The same approach was used to generate heterozygous *Rel^del^* flies. Flies heterozygous for both *Dif^del^* and *Rel^del^* were generated by crossing *Dif^del^*/*Dif^del^* to *Rel^del^*/*Rel^del^* flies. Control flies for +/*Dif^del^*;+/*Rel^del^* flies were generated by crossing the genetic background-matched control +/+ flies for *Dif^del^* and *Rel^del^*. *Dif^del^* and *Rel^del^* mutations were recombined into the *w^1118^* genetic background by backcrossing for eight generations, using the fluorescent DsRed eye marker to follow the mutations.

### TBI, early mortality, and lifespan

Flies were injured using a HIT device as described by Katzenberger et al. (2013, 2015b). In brief, vials containing 60 flies (1:1, female:male) at 0 to 7 or 20 to 27 days old were injured by 4 strikes at 5 min intervals with the spring deflected to 90°. Mixed-sex flies were used because females and males have equivalent early mortality (Katzenberger et al. 2013). Injured flies were transferred to vials containing CMYD or water and cultured at 25 °C. Water was used as an environmental perturbation to alter metabolic state without changing genetic background, allowing us to determine whether genotype-dependent differences in TBI outcomes are modulated by environmental context. Percent early mortality represents the percent of injured flies minus the percent of uninjured flies, examined at the same time, that died within 24 h. This procedure accounted for variation in intrinsic mortality among DGRP lines. In all cases, mortality of uninjured flies was <1%. The lifespan of adult mixed-sex flies that survived 24 h following TBI was determined using at least 265 flies per genotype. The number of surviving flies was counted each weekday until all flies had died. Flies were transferred to new vials approximately every 3 d.

### Quantitative reverse transcription PCR (qRT-PCR) analysis

Total RNA was extracted from 20 whole male flies using Trizol (Invitrogen). RNA purification was performed using the RNeasy Mini Kit and RNase-Free DNase (Qiagen). Males were used to avoid complexities due to differences in basal expression between females and males. For each sample, 1 µg of RNA was reverse transcribed using the iSCRIPT cDNA synthesis kit (Bio Rad). Quantitative PCR was performed using iTaq Universal SYBR Green SuperMix (Bio Rad) and the Bio Rad CFX96 Real-Time PCR Detection System. Biological replicates of each condition were performed in triplicate, and technical replicates of samples were performed in duplicate. Primer sequences are in Supplementary Table 1.

### Statistical analysis

Statistical analyses were performed using GraphPad Prism 8 and R version 4.5.1, with an arcsine square root transformation applied to early mortality data and a log_2_ transformation applied to gene expression data. Each vial, not individual flies, was treated as the unit of replication in all statistical analyses, such that variation among flies within a vial was not considered independent and vial-to-vial variance is appropriately reflected in the reported statistics. Effects of genetic background, age, and diet on early mortality and expression of individual genes were assessed using analysis of variance (ANOVA), omitting non-significant interaction terms, and with general linear (mixed) models; additionally, the effects of these factors on multivariate gene expression patterns were assessed using multivariate ANOVA (MANOVA).

## Results

### Genetic background, age, and diet modulate AMP expression before and after TBI

**Fig. 1.**
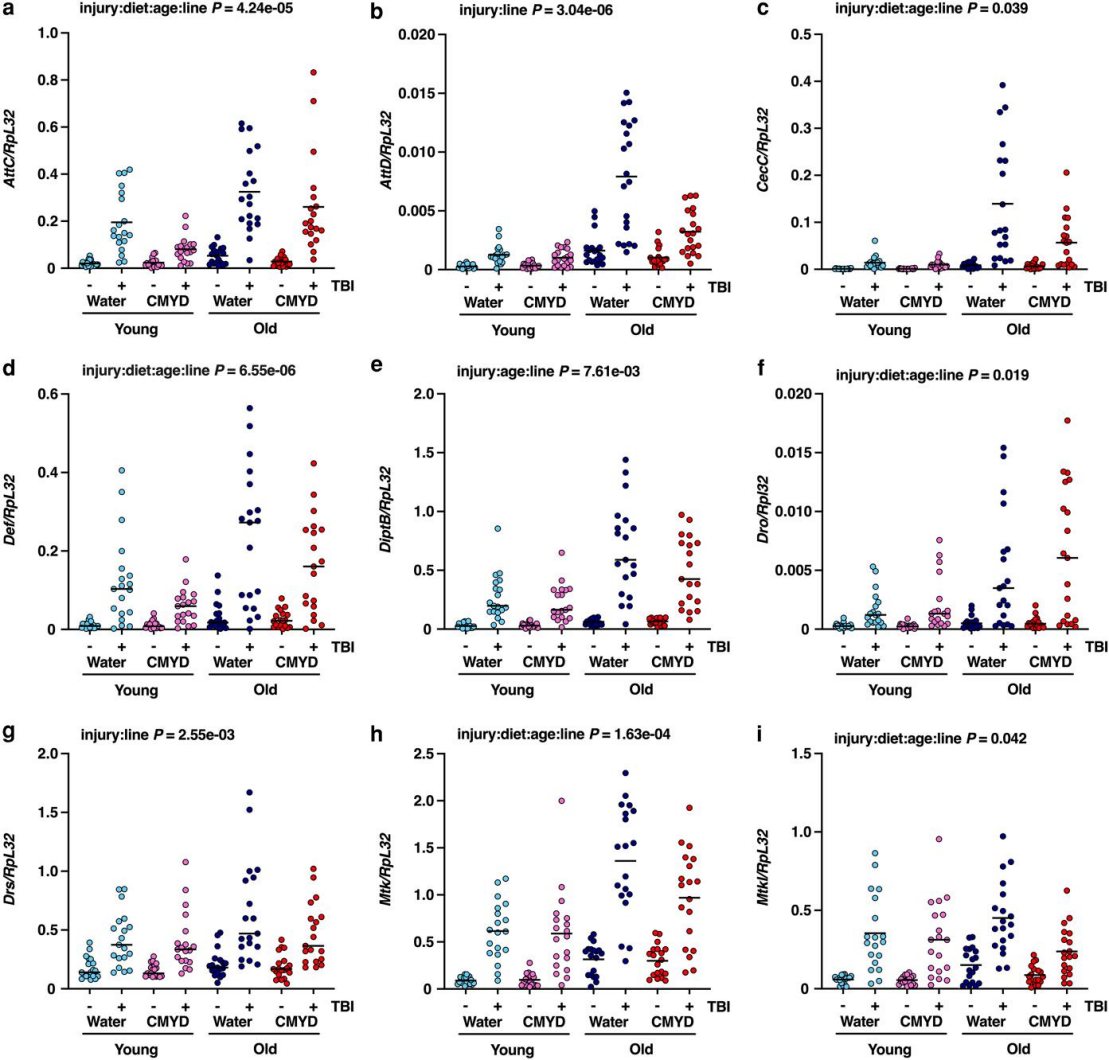
Genetic background, age, and diet influence injury-induced AMP expression. (a–i) Expression of nine AMP genes normalized to *RpL32* in 19 DGRP lines (dots), in uninjured (−) and injured (+) flies, under water, young (light blue); CMYD, young; water, old; and CMYD, old conditions. Each dot represents the average of three biological replicates. Horizontal bars indicate the average expression across the DGRP lines in a given context. Text in each panel represents the highest-level interaction effect involving injury detected in ANOVA, and the corresponding *P* value.

We previously showed in the standard laboratory strain *w^1118^* that TBI induces expression of most AMP genes at two ages (young: 0–7 d and old: 20–27 d) and under two diet conditions (water and CMYD) (Katzenberger et al. 2016). To test whether genetic background influences this response, we measured expression of nine AMP genes (*Attacin C*, *AttC*; *Attacin D*, *AttD*; *Cecropin C*, *CecC*; *Defensin, Def*; *Diptericin B*, *DiptB*; *Drosocin*, *Dro*; *Drosomycin*, *Drs*; *Metchnikowin*, *Mtk*; and *Metchnikowin-like*, *Mtkl*) in 19 randomly selected DGRP lines using qRT-PCR of whole-fly mRNA. In most lines, TBI increased expression of most AMPs across all four age-diet conditions (Fig. 1). Averaging across genes and DGRP lines, AMP expression was higher after injury (ANOVA; *F*_1,4100_ = 401.62, *P* < 1 × 10^−15^), higher in older flies (*F*_1,4100_ = 107.14, *P* < 1 × 10^−15^) and higher in flies fed water rather than CMYD (*F*_1,4100_ = 10.23, *P* = 1.39 × 10^−3^), suggesting that aging enhances TBI-induced activation of the Toll and Imd pathways and that fasting may further enhance this activation or carbohydrates may suppress it.

**Fig. 2.**
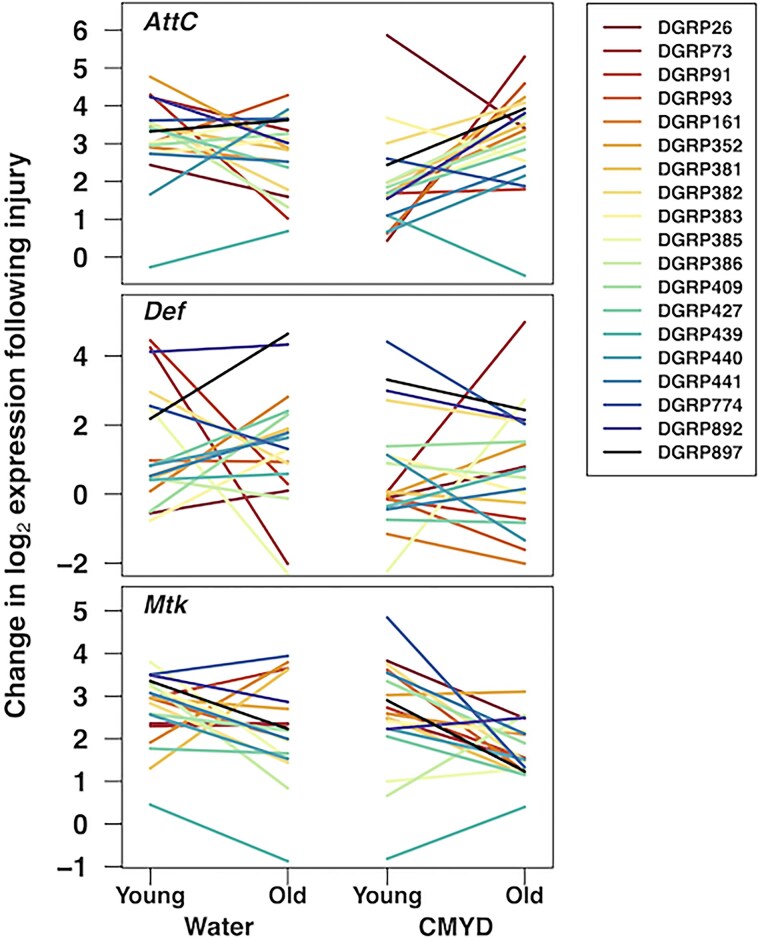
Injury-induced AMP expression is modulated by genetic background, age, and diet. The change in expression of *AttC*, *Def*, and *Mtk* between uninjured and injured flies for 19 DGRP lines under different age (young and old) and diet (water and CMYD) conditions. All three genes had a significant four-way interaction among injury, diet, age, and genetic background after a conservative correction for multiple testing (ANOVA; *AttC*: *F*_18,304_ = 3.04, *P* < 4.24 × 10^−5^; *Def*: *F*_18,304_ = 3.38, *P* < 6.55 × 10^−6^; *Mtk*: *F*_18,304_ = 2.80, *P* < 1.63 × 10^−4^).

An analysis of gene expression considering all genes and DGRP lines revealed a highly significant five-way interaction among injury, diet, age, gene, and line (ANOVA, *F*_144,2736_ = 1.72, *P* = 4.58 × 10^−7^), with interaction meaning that the effect of one factor on gene expression depends on the level(s) or one or more other factors. Interaction effects were evident both before (*F*_144,1368_ = 1.83, *P* = 5.03 × 10^−8^) and after injury (*F*_144,1368_ = 1.39, *P* = 2.35 × 10^−3^). As shown in Fig. 1, analyses of individual AMPs showed significant four-way interactions among injury, diet, age, and line for *AttC*, *CecC*, *Def*, *Dro*, *Mtk*, and *Mtkl*, three-way interactions among injury, age, and line for *DiptB*, and two-way interactions between injury and line for *AttD* and *Drs*. Applying a conservative correction for multiple testing (α = 0.05/(9 × 15), with 15 model terms tested for each of 9 genes), significant four-way interactions would remain for *AttC*, *Def*, and *Mtk*, two-way interactions between injury and line for *AttD*, *CecC*, *DiptB*, and *Dro*, and main effects of injury for *Drs* and *Mtkl*. Expression changes following injury in each context are shown in Fig. 2 for the three genes showing the strongest evidence for complex interactions, *AttC*, *Def*, and *Mtk*, illustrating the diversity in responses across DGRP lines. For example, among water-fed DGRP lines, TBI induced a larger increase in *AttC* expression in young flies than in old flies in some lines (*e.g*. DGRP91), whereas other lines showed the opposite age dependence (*e.g*. DGRP440). In addition, in certain lines, diet exerted opposite effects on TBI-induced *AttC* expression in young and old flies (*e.g*. DGRP439). Finally, in some DGRP lines under the water condition, diet differentially affected TBI-induced AMP expression (*e.g*. *Def* and *Mtk* in DGRP897). These results indicate that injury, genetic background, age, and diet, have complex effects on AMP expression.

Table 1 shows the percentage of variance in expression of each AMP gene attributable to each predictor. For most genes, the largest fraction of variance is explained by injury, followed by genetic background (DGRP line). Two exceptions are *AttD*, where age is the largest contributor to variance, and *Def*, where DGRP line is the largest contributor. These results reveal the complexity of AMP regulation, with each AMP influenced differently by interactions among injury, genetic background, age, and diet, acting through the Toll and/or Imd pathways.

**Table 1.** Percentage of variance in AMP gene expression explained by each predictor.

Predictor^a^	*AttC*	*AttD*	*CecC*	*Def*	*DiptB*	*Dro*	*Drs*	*Mtk*	*Mtkl*	Avg.
Line	12.95	10.50	9.15	**53**.**20**	9.35	28.33	24.81	17.45	18.56	20.48
Injury	**47**.**07**	19.88	**39**.**13**	4.37	**50**.**45**	**32**.**81**	**31**.**86**	**41**.**24**	**24**.**08**	**32**.**32**
Diet	2.36	2.31	1.53	0.12	0.14	0.00	0.59	0.49	1.94	1.05
Age	7.42	**29**.**14**	21.36	0.24	8.91	5.9	1.23	14.32	1.44	10.00
Line:injury	3.09	3.37	2.92	4.46	4.98	4.96	3.19	4.06	2.01	3.67
Line:diet	3.17	2.66	1.60	4.72	1.61	3.76	1.99	2.21	4.15	2.87
Injury:diet	0.24	0.41	0.46	0.38	0.19	0.08	0.08	0.16	0.07	0.23
Line:age	5.75	9.54	7.31	3.27	4.37	6.08	6.67	2.59	3.73	5.48
Injury:age	0.21	0.17	0.06	0.00	0.01	0.34	0.13	0.85	0.05	0.20
Diet:age	0.01	0.97	0.47	0.04	0.02	0.12	0.38	0.02	1.15	0.35
Line:injury:diet	1.34	1.45	1.18	1.75	1.16	0.97	0.93	0.61	1.20	1.18
Line:injury:age	1.77	1.07	1.61	1.15	1.80	1.09	0.84	0.90	3.37	1.51
Line:diet:age	1.18	1.82	1.65	2.20	1.49	2.15	2.20	1.03	2.84	1.84
Injury:diet:age	0.99	0.00	0.03	0.01	0.00	0.00	0.01	0.07	0.40	0.17
Line:injury:diet:age	1.90	0.63	1.05	4.01	0.85	1.33	0.90	1.99	3.16	1.76
Residual	10.54	16.08	10.48	20.08	14.67	12.08	24.19	11.99	31.85	16.88

^a^For each gene, the predictor with the largest contribution is shown in bold.

### Genetic background, age, diet, and their interactions affect early mortality following TBI

**Fig. 3.**
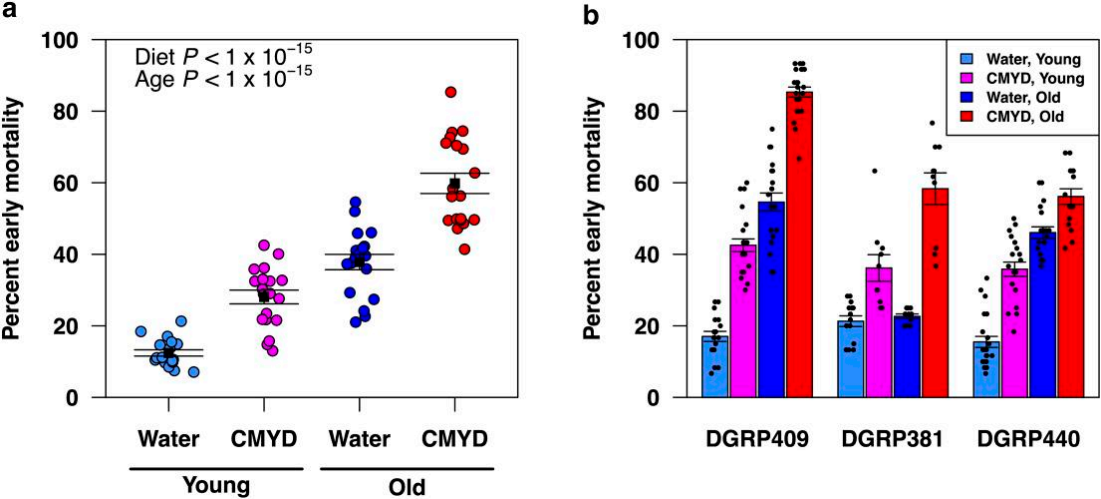
Genetic background, age, and diet influence early mortality following TBI. Early mortality in (a) 19 DGRP lines (dots) and (b) three selected DGRP lines under the indicated age-diet conditions. Black points in (a) indicate the average early mortality across the DGRP lines. In (a) and (b), error bars indicate mean ± SEM. *P*-values are shown for the average effects of diet and age (see text for details).

To assess effects of genetic background, age, and diet on TBI outcomes, we determined early mortality for the 19 DGRP lines under the four age-diet conditions. Table 2 shows that the percentage of variance in early mortality attributable to each predictor. Age had the greatest effect, followed by diet and genotype. Consistent with previous findings (Katzenberger et al. 2015a, 2016, 2023), average early mortality across DGRP lines was higher for flies fed CMYD vs water (linear model: *t* = 31.33*, P* < 1 × 10^−15^) and in old vs young flies (*t* = 49.49*, P* < 1 × 10^−15^), with no significant interaction between these effects (*t* = −1.53, *P* = 0.13) (Fig. 3a). Furthermore, effects of age and diet on early mortality varied with genetic background (ANOVA: three-way interaction *F*_18,1321_ = 8.56, *P* < 1 × 10^−15^). For example, in DGRP409, early mortality increased with both age (*t* = 21.18, *P* < 1 × 10^−15^) and diet (*t* = 15.25, *P* < 1 × 10^−15^), with no interaction; in DGRP381 there was a synergistic interaction between old age and CMYD diet (*t* = 3.49, *P* = 1.20 × 10^−3^); and in DGRP440 there was an antagonistic interaction between old age and CMYD diet (*t* = −3.44, *P* = 9.51 × 10^−4^) (Fig. 3b). These findings indicate that differences in genetic background, age, and diet and their interactions contribute significantly to variation in TBI outcomes.

**Table 2.** Percentage of variance in early mortality explained by each predictor.

Predictor	Variance (%)
Line	10.32
Diet	21.08
Age	50.41
Line:diet	3.02
Line:age	3.50
Diet:age	0.11
Line:diet:age	1.29
Residual	10.26

### AMP expression correlates more strongly with early mortality in flies pre-injury than post-injury, and the correlation is more positive in young flies than in old flies

**Fig. 4.**
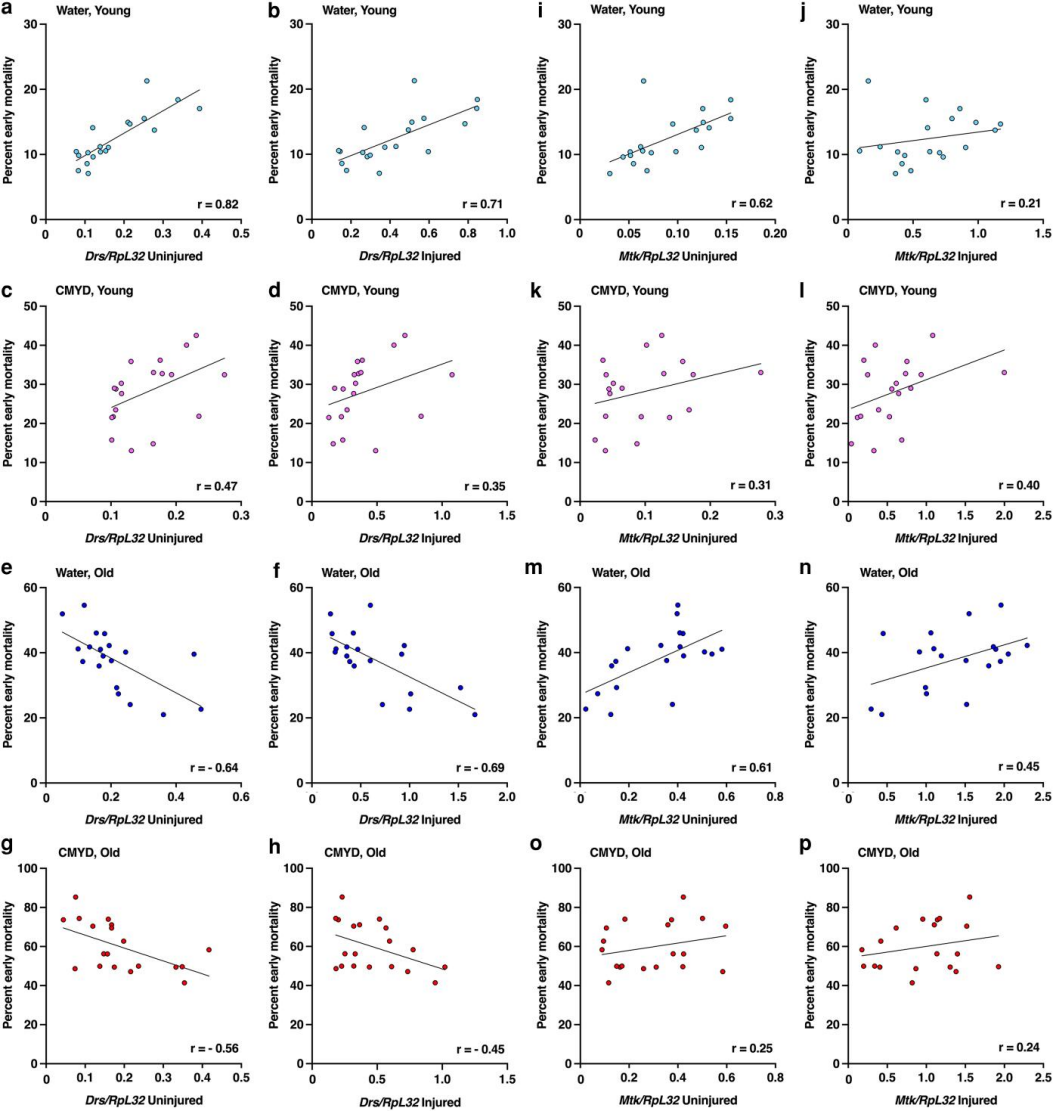
*Drs* and *Mtk* expression correlates with early mortality after TBI in the same way in young flies, but in opposite ways in old flies. Untransformed (a–h) *Drs* and (i–p) *Mtk* expression normalized to *RpL3*2 (from Fig. 1) are plotted against early mortality in 19 DGRP lines (dots) (from Fig. 3). Pearson correlation coefficients (*r*) and regression lines are shown for each condition. Diet, age, and injury status are indicated for each panel.

**Fig. 5.**
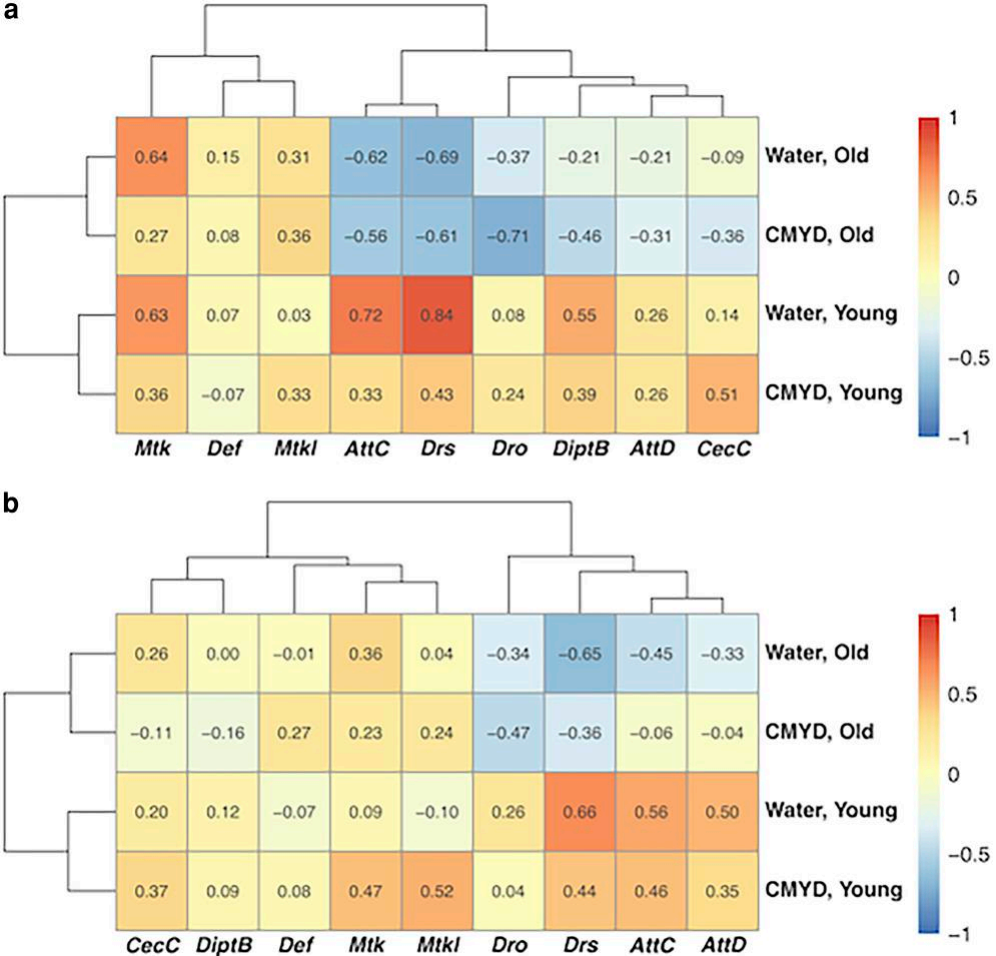
AMP expression shows a stronger correlation with early mortality in uninjured than in injured flies and a more positive correlation in young than in old flies. Each cell shows the correlation between early mortality and transformed gene expression (see Statistical analysis section of the Materials and methods) for a particular gene (columns) and context (rows) for (a) uninjured flies and (b) injured flies, with correlation strength also indicated by color (legend). Dendrograms show hierarchical clustering of rows and columns.

To assess the relationship between AMP expression and early mortality, we compared the expression data in Fig. 1 with the mortality data in Fig. 3. The strength and direction of correlations between AMP expression and early mortality varied widely across different AMP genes and age-diet conditions, as shown by analyses of primary data for *Drs* (Fig. 4a–h) and *Mtk* (Fig. 4i–p) as well as transformed data for all nine AMPs (Fig. 5). Data quality is supported by hierarchical clustering that groups *Mtk* with *Mtkl* genes with closely related functions (Tattikota et al. 2020). Averaging across conditions, absolute correlations were stronger for basal (pre-injury) (Fig. 5a) than post-injury expression (Fig. 5b) (paired *t*-test, *t* = 2.61, *df* = 8, *P* = 0.031). Average correlations were similar in water-fed and CMYD-fed flies (0.09 vs. 0.08, paired *t* = 0.32, *df* = 17, *P* = 0.75), and more positive in young flies than in in old flies (0.31 vs. −0.14, paired *t* = 4.18, *df* = 17, *P* = 6.30 × 10^−4^). These patterns indicate that (i) basal expression prior to injury can be more predictive of early mortality than TBI-induced expression, and (ii) AMPs promote TBI-triggered early mortality in young flies but protect against it in old flies. Based on their known properties, including whether they are regulated by the Toll or Imd pathway, there is no clear distinction between AMPs whose correlations change with age and those that remain stable (Hanson et al. 2025).

### A null allele of *Dif* increases the lifespan of uninjured and injured flies

**Fig. 6.**
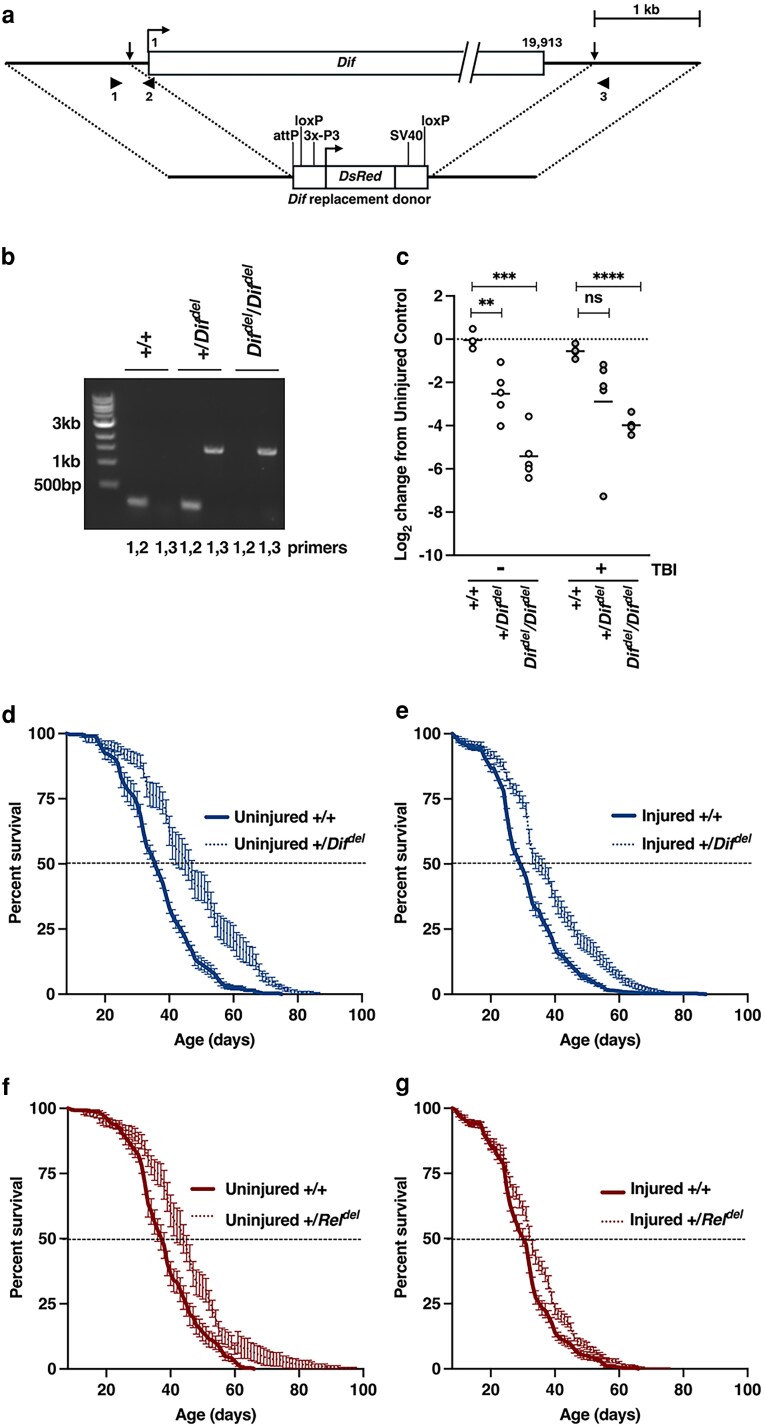
Lifespan is extended in both uninjured and injured heterozygous *Dif^del^* mutants but only in uninjured *Rel^del^* mutants. (a) Diagram of the wild-type *Dif* genomic locus and the *Dif* replacement donor used to generate the *Dif^del^* allele. Horizontal arrows indicate transcription start sites, vertical arrows indicate sites targeted by sgRNAs for cleavage by Cas9, numbered arrowheads indicate the direction and identity of primers used for PCR in panel b, and dotted lines indicate ∼1 kb homology arms on either side of the *DsRed* gene that were used for homology directed repair. *DsRed* is described in more detail in Gratz et al. (2014). (b) PCR analysis of the *Dif* locus using genomic DNA from +/+, +/*Dif^del^*, and *Dif^del^*/*Dif^del^* flies in uninjured (−) and injured (+) flies. PCR primers indicated for each lane are diagrammed in panel a. c) qRT-PCR of *Dif* expression normalized to *RpL32* from +/+, +/*Dif^del^*, and *Dif^del^*/*Dif^del^* flies collected from uninjured (−) and injured (+) flies. Unpaired two-tailed t-tests compared expression between control and mutant flies, ns = not significant, **P < 0.01, ***P < 0.001, and ****P < 0.0001. Percent survival of (d) uninjured and (e) injured control (+/+) and +/*Dif^del^* flies and (f) uninjured and (g) injured control (+/+) and +/*Rel^del^* flies. Horizontal lines indicate the median lifespan. Error bars indicate the SEM.

To test whether the Toll pathway directly contributes to early mortality after TBI, we used CRISPR-Cas9 gene editing to generate a null allele of *Dif* (*Dif^del^*) (Fig. 6a), analogous to our previously generated *Rel^del^* allele for the Imd pathway (Swanson et al. 2020b). We created a new *Dif* mutant line to ensure a uniform genetic background between control and *Dif* mutant flies, as genetic background strongly influences early mortality (Fig. 3). The *Dif* coding region was deleted in the control line and replaced with *DsRed*, encoding a red fluorescent protein used to track the *Dif^del^* genotype (Fig. 6a). PCR of genomic DNA confirmed the genotypes of +/*Dif^del^* and *Dif^del^*/*Dif^del^* flies (Fig. 6b). qRT-PCR of whole fly RNA showed a 2- to 4-fold reduction in *Dif* mRNA expression in uninjured and injured +/*Dif^del^* flies and complete loss of expression in *Dif^del^*/*Dif^del^* flies (Fig. 6c), confirming that *Dif^del^* is a null allele.

To assess general effects of *Dif^del^* and *Rel^del^* mutations on fly physiology, we measured the lifespan of mixed-sex control and heterozygous mutant flies under uninjured and injured conditions. *Dif^del^* heterozygotes lived longer than genetic background-matched controls under both uninjured (Kaplan–Meier rank sum test, *P* < 0.0001; Fig. 6d) and injured conditions (*P* = 0.0002; Fig. 6e). In contrast, *Rel^del^* heterozygotes had an extended lifespan only when uninjured (*P* = 0.002; Fig. 6f), with no difference observed after injury (*P* = 0.56; Fig. 6g). For both mutant heterozygotes, median lifespan was shorter in injured flies than uninjured flies (+/*Dif^del^*: 39.4 vs. 47.02 d; +/*Rel^del^*: 35.3 vs 44.5 d), indicating that TBI exerts lasting effects even when Toll and Imd signaling are reduced. Overall, these findings indicate that reducing Toll and Imd signaling does not compromise fly physiology.

### *Dif^del^* and *Rel^del^* mutations reduce early mortality after TBI in a genetic background-, age-, and diet-dependent manner

**Fig. 7.**
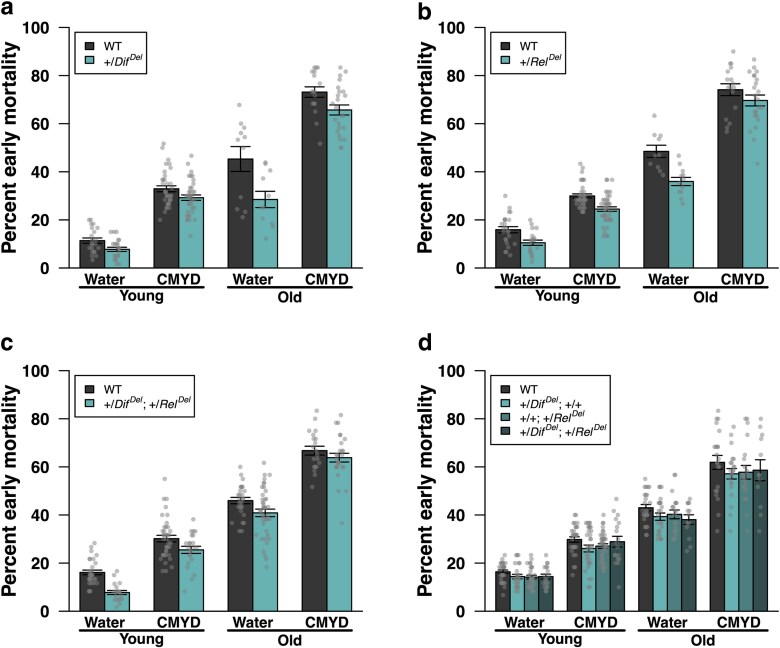
Genetic background, age, and diet affect how *Dif^del^* and *Rel^del^* mutations influence early mortality following TBI. (a) Heterozygous deletion of *Dif* was associated with reduced mortality, particularly in old flies. (b) Heterozygous deletion of *Rel* was associated with reduced early mortality, with no interaction with age or diet. (c) Heterozygosity for both deletions was associated with reduced early mortality, particularly in young flies and flies fed water. (d) In the *w*^1118^ background, the heterozygous deletions show positive epistasis for early mortality, without interactions with diet or age. Error bars represent SEM. See text for details.

To test whether Toll and Imd signaling directly influence TBI outcomes, we examined effects of *Dif^del^* and *Rel^del^* mutations on early mortality (Fig. 7). Heterozygous deletion of *Dif* was associated with reduced early mortality, particularly in old flies (linear model interaction: *t* = −2.17, *P* = 0.031, Fig. 7a). Heterozygous deletion of *Rel* was also associated with reduced early mortality, but with no significant interactions with diet or age (linear model main effect: *t* = −5.58, *P* = 8.63 × 10^−8^, Fig. 7b). Heterozygosity for both deletions reduced early mortality to a greater degree in flies fed water (linear model interaction: *t* = 2.28, *P* = 0.024, Fig. 7c) and in young flies (linear model interaction: *t* = 2.15, *P* = 0.033, Fig. 7c). In the *w^1118^* background, with four possible genotypes, there was a significant positive epistasis between the *Dif* and *Rel* heterozygous deletions (linear model interaction: *t* = 2.39, *P* = 0.018, Fig. 7d), which did not depend on diet or age.

**Fig. 8.**
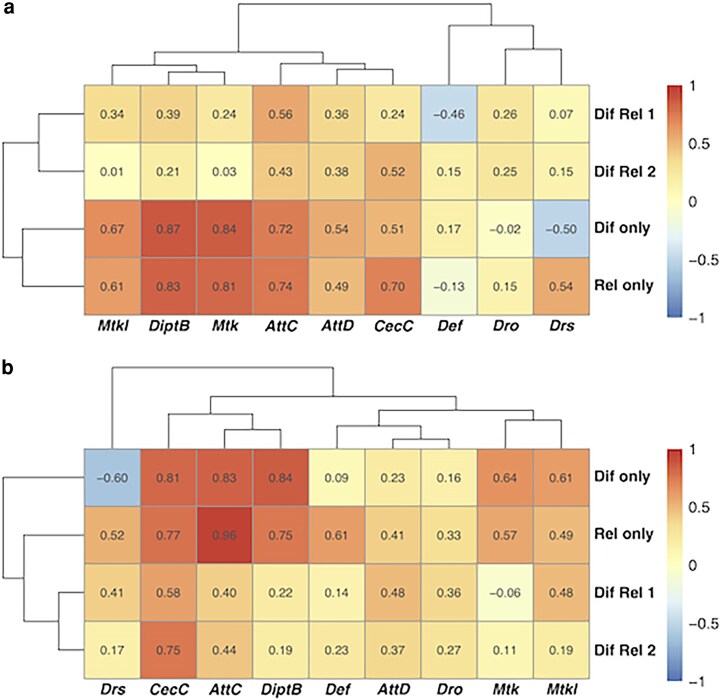
Changes in AMP expression cause by *Dif^del^* and *Rel^del^* mutations are correlated with early mortality following TBI. Each cell shows the correlation between early mortality and gene expression for a particular gene (columns) and genetic context of *Dif* and *Rel* mutations (rows) for uninjured flies (a) and injured flies (b), with correlation strength also indicated by color (legend). The genetic contexts match those shown in the panels of Fig. 7. Dendrograms show hierarchical clustering of rows and columns.

To test whether effects of *Dif^del^* and *Rel^del^* mutations on early mortality after TBI (Fig. 8) were mediated by changes in AMP expression, we measured transcript levels of the nine AMP genes by qRT-PCR in mutant and control flies, before and after injury, across the four age-diet conditions, and in multiple genetic backgrounds. Data quality is supported by hierarchical clustering that groups the two lines heterozygosity for both *Dif^del^* and *Rel^del^* (Dif Rel 1 and Dif Rel 2). Averaging across AMP genes and conditions, gene expression was consistently lower in the presence of *Dif^del^* or *Rel^del^* (linear mixed model with random effect of gene, *t* = −9.72, *P* < 1 × 10^−15^, Fig. 8). However, we found that *Dif^del^* and *Rel^del^* affected AMP expression patterns in a context-dependent fashion. *Dif^del^* interacted with diet (MANOVA: *F*_9,40_ = 2.66, *P* = 0.016) and injury (*F*_9,40_ = 2.33, *P* = 0.032) in influencing AMP expression; similarly, *Rel^del^* interacted with genotype and diet (*F*_9,40_ = 2.41, *P* = 0.027). Effects of heterozygosity for both *Dif^del^* and *Rel^del^* on AMP expression interacted with diet (*F*_9,47_ = 4.16, *P* = 5.39 × 10^−4^), age (*F*_9,47_ = 3.53, *P* = 2.08 × 10^−3^), and injury (*F*_9,47_ = 3.87, *P* = 9.95 × 10^−4^) (Dif Rel 1 in Fig. 8). Finally, in the *w^1118^* genetic background, there was an interaction between *Dif^del^* and *Rel^del^* (epistasis; *F*_9,110_ = 15.59, *P* = 3.87 × 10^−16^), and both *Dif^del^* and *Rel^del^* interacted with age (*Dif*: *F*_9,110_ = 2.62, *P* = 8.80 × 10^−3^; *Rel*: *F*_9,110_ = 2.11, *P* = 0.034) (Dif Rel 2 in Fig. 8). Together, these results indicate positive epistasis between *Dif* and *Rel* in regulating TBI-induced early mortality, and complex, context-dependent epistasis in controlling AMP expression.

### Effects of NF-κB mutations on AMP expression correlate with early mortality after TBI

We next examined whether changes in AMP expression were correlated with early mortality following TBI across contexts, considering pre-injury (Fig. 8a) and post-injury (Fig. 8b) patterns separately. In other words, we sought to determine whether expression of a given AMP gene, influenced by diet, age, and *Dif/Rel* genotype, was predictive of early mortality. We found that these correlations were typically positive: 32 out of 36 cases for uninjured flies (Fig. 8a) and 34 out of 36 cases for injured flies (Fig. 8b). For each genetic background and injury condition (8 groups), randomization tests (10,000 simulations each) showed that the average correlation was significant (*P* < 0.01), except in the case of uninjured +/*Dif^del^*; +/*Rel^del^* flies from the first genetic background (Dif Rel 1 in Fig. 8), where the result was marginally nonsignificant (*P* = 0.079). These data indicate that reductions in AMP expression caused by deletion of *Dif* or *Rel* result in proportional reductions in early mortality following TBI. Although correlative analyses across age and diet contexts suggest that higher basal AMP expression is protective in older flies (Fig. 5), effects observed in *Dif* and *Rel* mutants likely reflect the fact that these NF-κB factors regulate many gene targets beyond AMPs, such that reduced early mortality in these mutants may result from broader transcriptional changes rather than AMP reductions per se (Swanson et al. 2020b).

## Discussion

### Implications for mammalian models and human TBI

Our findings indicate that variation in TBI outcomes, even among individuals with comparable injuries, is due to injury-, genotype-, age-, and diet-dependent differences in activation of innate immunity gene expression. Consequently, mammalian studies conducted in a single genetic background, age, or dietary conditions are unlikely to capture the full range of neuroinflammatory responses and behavioral and physiological outcomes observed in humans. Each factor (injury, genetic background, age, and diet) independently contributes to variation in innate immunity gene expression (Table 1) and early mortality after TBI (Table 2), with interactions among these factors further amplifying this variation. Unexpectedly, the relationship between AMP expression and early mortality reversed with age, with generally positive correlations in young flies and negative correlations in old flies (Figs. 4 and 5). This age-dependent reversal suggests that innate immune effectors may have fundamentally different, and even opposing, effects on TBI outcomes at different life stages in mammals, including humans, raising the possibility that immune-modulating therapies could have opposite effects in younger and older individuals.

These conclusions help reconcile several long-standing inconsistencies in the TBI literature. In mammalian models, injury paradigms that are nominally similar often yield divergent neuroinflammatory profiles and outcomes across laboratories, strains, ages, and husbandry conditions (Cortes and Pera 2021; Islam et al. 2021; Radabaugh et al. 2023; Shannon et al. 2024; Kislik et al. 2025). Our results suggest that such variation is not simply experimental noise, but instead reflects predictable differences in how innate immune pathways are engaged by injury in distinct biological contexts. For example, age-dependent changes in inflammatory signaling, strain-specific differences in resting immune activity, and diet-induced alterations in metabolism and immune function have each been shown to influence TBI pathology, yet are rarely examined in combination. The strong context dependence we observe implies that conclusions drawn from a single strain, young adult, *ad libitum*-fed animal models may generalize poorly to older individuals, metabolically altered states, and genetically diverse human populations. More broadly, our findings argue that innate immune activation should be viewed as a multidimensional modifier of TBI outcomes rather than a uniform response to injury, and they underscore the need for experimental designs and therapeutic strategies that explicitly incorporate genetic diversity, age, and metabolic state to better reflect the heterogeneity of TBI in humans.

### Basal innate immune gene expression predicts TBI outcomes

Our data suggest that basal expression of innate immune genes is a stronger predictor of TBI outcomes than post-injury activation (Figs. 4 and 5). Similarly, aging and pre-existing environmental factors that affect basal expression influence TBI outcomes in mammals (Houle and Kokiko-Cochran 2022). Pre-injury activation of TLR pathways by bacterial lipopolysaccharide (LPS) or by polyinosinic-polycytidylic (poly I:C, a mimic of viral double-stranded RNA) can be neuroprotective after TBI (Kadowaki et al. 2001; Turner et al. 2017; Eslami et al. 2020), whereas infection with the parasite *Toxoplasma gondii* worsens TBI outcomes (Baker et al. 2024). Evidence from human studies indicate that cytokine expression following TBI is also predictive of outcomes. For instance, elevated levels of interleukin (IL-6) and TNF-α are associated with worse clinical outcomes (Malik et al. 2023), IL-6 predicts six-month mortality (Cáceres et al. 2025), IL-1β is associated with a higher risk of post-traumatic epilepsy (Diamond et al. 2015), and a broad cytokine profile can predict post-traumatic depression (Juengst et al. 2015; Di Battista et al. 2026). Together, these findings underscore the importance of considering both basal and post-injury cytokine expression in predicting TBI outcomes.

### Limitations of the study

A limitation of this study is that AMP expression was measured in whole flies. In adults, AMPs are expressed in multiple tissues and cell types, including fat body (abdomen and head), gut and tracheal barrier epithelia, hemocytes, and glia (Lee et al. 2024). Consequently, AMP expression specifically relevant to early mortality after TBI may be restricted to a subset of these tissues/cells and is masked in bulk measurements, potentially leading to misinterpretation of the relationship between AMP levels and early mortality. Similarly, the *Dif^del^* and *Rel^del^* mutations that reduced early mortality were present in all adult cells (Fig. 7). We hypothesize that innate immune signaling relevant to early mortality occurs primarily in brain glia. Supporting this, activation of the Imd pathway or misexpression of AMPs in glia leads to neurodegeneration (Petersen et al. 2012, 2013; Cao et al. 2013; Nukala et al. 2023), a long-term outcome in the fly TBI model, while reducing Imd pathway signaling in glia diminishes neurodegeneration and extends lifespan (Kounatidis et al. 2017). We found that heterozygous *Dif^del^* and *Rel^del^* mutations also increased lifespan (Figs. 6d and f), indicating that although the Toll and Imd pathways were inhibited systemically, physiological effects were largely manifested in glia. To directly address possible cell type-specific effects, future studies will incorporate cell type-specific knockdown of *Dif* and *Rel* as well as single-cell expression analyses.

## Supplementary Material

jkag060_Supplementary_Data

## Data Availability

The authors affirm that all data necessary for confirming the conclusions of the article are present within the article, figures, and tables. Data used in the figures and tables are provided in Supplementary Data 1–6. Supplemental material available at G3 online.
