# Supplementary material for: Genetic variation in innate immune gene expression influences mortality after traumatic brain injury in Drosophila
Source: G3 (Bethesda). 2026 Mar 13;16(5):jkag060. doi: 10.1093/g3journal/jkag060 (PMC13148387; doi:10.1093/g3journal/jkag060)
Supplement: jkag060_Supplementary_Data [file jkag060_supplementary_data.zip › Table_S1_G3-2026-406560.docx]

**Table S1. Primers for PCR**

| **Gene** | **Forward** | **Reverse** |
| --- | --- | --- |
| *Dif* – Figs. 6a and b | CACCAATAAGCAACCCAAAGG (primer 1) | CCGTTTATTCCACTTGTGTTAAG (primer 2) |
| *Dif* – Figs. 6a and b | CACCAATAAGCAACCCAAAGG (primer 1) | GTCATAGAGCCACCACTGAA (primer 3) |
| *Dif –* Fig. 6c | CAGTTTGCTACGACCGGAGAGCTA | GAATATCCGCCAGTTGCAGAGTGC |
| *AttC* | CTGCACTGGACTACTCCCACATCA | CGATCCTGCGACTGCCAAAGATTG |
| *AttD* | CATTCAGAGCGGCGTTATTG | CGGTCACCAAGGGAGTTTAT |
| *CecC* | TCAGCATTGGACAATCGGAAG | CAGTCCTTGAATGGTTGCATC |
| *Def* | CCAGAGGATCATGTCCTGGTGCAT | ACTTGGAGAGTAGGTCGCATGTGG |
| *DiptB* | AGGATTCGATCTGAGCCTCAACGG | TGAAGGTATACACTCCACCGGCTC |
| *Dro* | TGAGTCAGGTGATCCTCGAT | GCTGCTTGCTTGCGTTT |
| *Drs* | AGTACTTGTTCGCCCTCTTCGCTG | CCTTGTATCTTCCGGACAGGCAGT |
| *Mtk* | CATCAATCAATTCCCGCCACCGAG | AAATGGGTCCCTGGTGACGATGAG |
| *Mtkl* | TTCCTGGGCGAGGCAAG | TGGAGATGGCCTCATATCGAAAG |
| *RpL32* | GACGCTTCAAGGGACAGTATCTG | AAACGCGGTTCTGCATGAG |
